# Comparison between two scoring methods to assess tail damage of docked pig carcasses during postmortem inspection in Ireland

**DOI:** 10.1002/vro2.66

**Published:** 2023-08-21

**Authors:** Roberta Maria D'Alessio, Conor G. McAloon, Laura Ann Boyle, Alison Hanlon, Keelin O'Driscoll

**Affiliations:** ^1^ Department of Pig Development, Teagasc Animal & Grassland Research and Innovation Centre Moorepark, Fermoy, Co. Cork Ireland; ^2^ UCD Veterinary Sciences Centre University College Dublin, Belfield Dublin Ireland

## Abstract

**Background:**

Tail inspection in the abattoir is a tool to help determine the welfare status of pigs. However, methodologies vary widely. Moreover, meat inspection is moving from palpation and incision towards visual‐only (VIS) examination. This study investigated whether a VIS examination was sufficient to detect tail damage compared to handling (HAND), which ensures examination of all aspects of the tail.

**Method:**

The severity of tail skin damage (0 [undamaged] – 4 [partial/full loss of tail]) and presence/absence of bruises was scored using both methods after scalding/dehairing of 5498 pig carcasses.

**Results:**

There was a good relationship between methods when evaluating tail skin damage (sensitivity, 82.48%; specificity, 99.98%; accuracy, 98.98%; correlation *ρ* = 0.84). The results were similar for the presence of bruises (sensitivity, 74.98%; specificity, 99.09%; accuracy, 89.94%; correlation *ρ* = 0.79). However, the percentage of tails classified as undamaged was higher using VIS (69.9%) than HAND (63.55%) examination. Conversely, VIS detected fewer mild lesions (score 1 – 13.64%; score 2 – 11.73%) than HAND (score 1 – 15.21%; score 2 – 15.53%). A higher percentage of bruises was detected using HAND than VIS (37.96% vs. 29.03%).

**Conclusions:**

Visual evaluation is a valid alternative to handling evaluation of carcass tail damage and bruising.

## INTRODUCTION

In the European Union (EU), Regulation 625/2017 states that abattoirs have a role to play in determining compliance with animal welfare legislation. Additionally, the European Food Safety Authority scientific opinion on pig welfare stated that ‘Monitoring tail lesions, carcass condemnation, lung lesions in rearing pigs at slaughter should be implemented to identify herds with diverse welfare consequences, thereby enabling guidance for the implementation of preventive and mitigation measures’.^1^ Indeed, lesions such as bites, scars or necrosis located on the tail are considered ‘iceberg indicators’ of welfare problems on pig farms,^2^ as such lesions are indicative of tail biting behaviour. Tail biting is a common behavioural disorder in intensively produced pigs, affecting animal health and welfare and the economy of farms.^3^, ^4^ The origin of this disorder is multifactorial,^5^, ^6^ including lack of enrichment, environmental discomfort, health problems and competition for resources.^3^, ^7^, ^8^, ^9^ It has a range of serious consequences, such as stress, pain and infections in bitten pigs,^7^ with reduced daily earnings for producers.^10^ A prevalence of only 0.86% of severe tail lesions has potential to cause up to a 15.1% reduction in the mean annual profit of the farm.^11^ Moreover, associations between tail damage and related lesions leading to carcase trimming and slaughterhouse condemnation are described.^7^, ^8^, ^11^, ^12^, ^13^, ^14^

The most commonly used strategy to reduce the risk of tail biting is to dock the tail. The EU has developed several initiatives to reduce this widespread practice. Council Directive 2008/120/EC states that it should only be allowed if other measures to prevent tail biting have failed.^15^, ^16^ A significant problem is that there is not enough information with regard to the prevalence of tail biting and docking at the national level. It has been proposed by the European Commission's Directorate General for Health and Food Safety (DG‐SANTE) that tail lesions should be assessed at the slaughterhouse and that for benchmarking purposes, the proportion of intact tails should be used,^17^ along with data regarding the severity of any lesions.

Tail lesions are easily identified and recorded during visual (VIS) postmortem examination because their visibility increases after scalding and dehairing of carcases.^2^ Commission Regulation (EU) No. 219/2014 allows the use of palpation or incision of swine carcases only when the veterinarians or meat hygiene inspectors identify some clinical signs and lesions that may indicate a possible risk to public health, animal health or animal welfare. Otherwise, the postmortem inspection of swine should be VIS.^18^ Indeed, a VIS meat inspection protocol was comparable to the traditional method that used manipulation and was deemed feasible for adoption.^19^ With regard to tail scoring, a potential limitation of VIS inspection is the possibility of confusing tail hair burns with minor injuries derived from tail biting; some hairs remain even after scalding, which can affect the validity of the examination.^13^ Length of the tail, height of the observer and the position of the tail on the line are other limitations of VIS examination because they can prevent the observer from examining the full extent of the tail. Manipulation/palpation may also be necessary to detect any change in tissue texture and the shape of the tail tip.^13^ On the other hand, tail manipulation will not always be possible in a practical situation, for instance, where the line speed is very fast.

With regard to severity scoring, several systems have been proposed and validated in commercial abattoirs,^2^ normally using a five‐ or six‐point scale, from 0 (perfect) to a top score indicating severe damage. To date, no scoring system has considered the presence of bruises independent of other damage to the pigs’ tail, such as skin breakage. This is the case even though biting may also cause bruises, even if the skin is not damaged.^20^ Given that bruises are possibly easier to detect than minor skin breaks, scoring bruising separate from bite marks could mitigate the inability to handle the tails and thereby improve the accuracy of VIS scoring systems in detecting the effects of tail biting.

This study investigated whether a VIS examination of pigs’ tails was sufficient to detect skin damage and the presence of bruises when compared to examination by handling (HAND), which is the conventional method. We hypothesised that a VIS postmortem examination was a valuable substitute for detecting tail damage and the presence of bruises on pigs’ tails if HAND is not possible. Additionally, we hypothesised that scoring bruises only could help identify mild tail skin damage using VIS examination.

## MATERIALS AND METHODS

The study population (*n* = 5498) comprised pigs slaughtered at four abattoirs in the Republic of Ireland, visited between February 2020 and June 2022. This study was part of a larger study evaluating a tail biting risk assessment protocol.

Study farms were selected by contacting pig producers who had a tail biting risk assessment carried out by private veterinary practitioners (PVPs) during the previous month via the Animal Health Ireland (AHI) PigHealthCheck programme (https://animalhealthireland.ie/programmes/pig‐healthcheck/). The AHI is a partnership between private sector organisations, businesses in the agri‐food sector and the Irish Department of Agriculture, Food and the Marine. It co‐ordinates the provision of tail biting risk assessments free of charge to pig producers by trained PVPs. The study authors were informed that a risk assessment was completed by the PigHealthCheck co‐ordinator when a report was uploaded to the AHI database. A total of 22 farms were recruited. Once pig producers granted permission to inspect their pigs, the next batch of pigs sent to the abattoir from their farm was inspected post slaughter. During each visit, a trained observer collected the following data from all animals in the batch: sex, tail skin damage score, presence/absence of bruises on the tail and whether there was severe tail loss with healing.

### Tail lesion scoring

Data collection took place after scalding/dehairing operations using two methods. The first method was a VIS examination, whereby the observer inspected the tail without touching it and recorded the score while directly facing the tail without manipulating the carcase. The height at which the carcass tails could be evaluated was dependent on the abattoir and tail length, so the VIS could be either of the dorsal or the ventral aspect of the tail. This method was always carried out first because this was the method under investigation, and we did not want to bias the scores by carrying out the more thorough HAND method prior to it.

Immediately after VIS, the tails were handled (HAND) and scored again based on what was detected. The observer manipulated the tail so that all aspects (lateral, dorsal and ventral sections) could be inspected more closely than when using VIS. At the beginning of the study, one author was trained in using both systems by the developer of the scoring method. During this period, intra‐observer reliability was evaluated. Afterwards, all tail examinations were only performed by one author. A similar distance was kept between the observer and the carcasses while performing both VIS and HAND observations.

**FIGURE 1:**
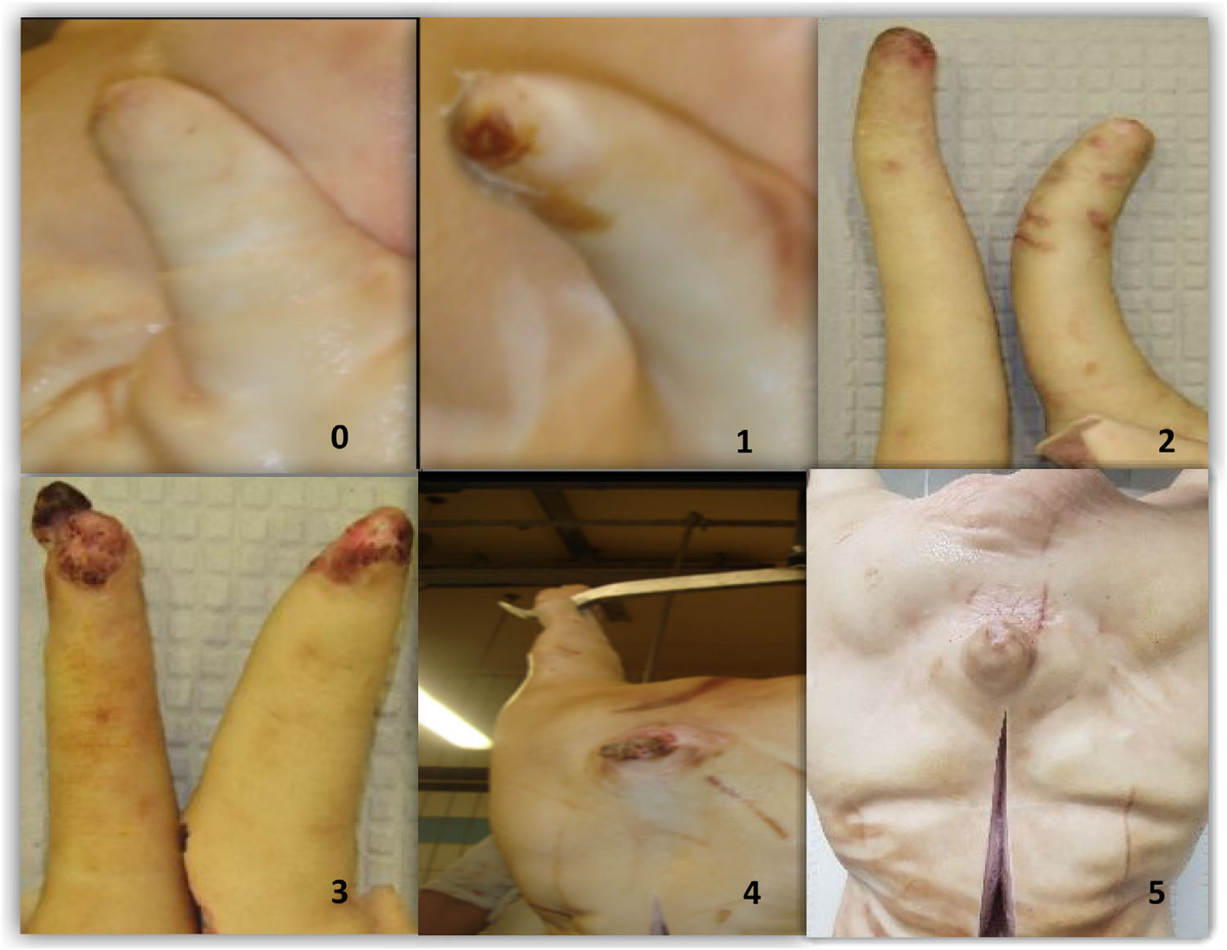
Adapted tail skin damage scoring system. 0: no evidence of tail biting; 1: minor skin damage to the tail tip without teeth marks; 2: evidence of teeth marks, with breakage to the skin and redness; 3: breakage of the skin with redness and swelling; 4: fresh partial or complete tail loss, an open wound on the tail accompanied by pus or necrotic tissue; 5: severe tail loss with healing.

Tail skin damage was classified into five levels according to severity: 0—no evidence of tail biting; 1—minor skin damage to the tail tip without teeth marks; 2—evidence of teeth marks, with breakage to the skin and redness; 3—breakage of the skin with redness and swelling; 4—fresh partial or complete tail loss, an open wound on the tail accompanied by pus or necrotic tissue. Scores 1 and 2 were classified as mild lesions, score 3 was classified as a moderate lesion and score 4 was classified as a severe tail lesion. Additionally, we recorded severe tail loss with healing (score 5), defined as presence of scar tissue after tail loss, because it represented a history of tail biting on the farm (Figure 1).

**FIGURE 2:**
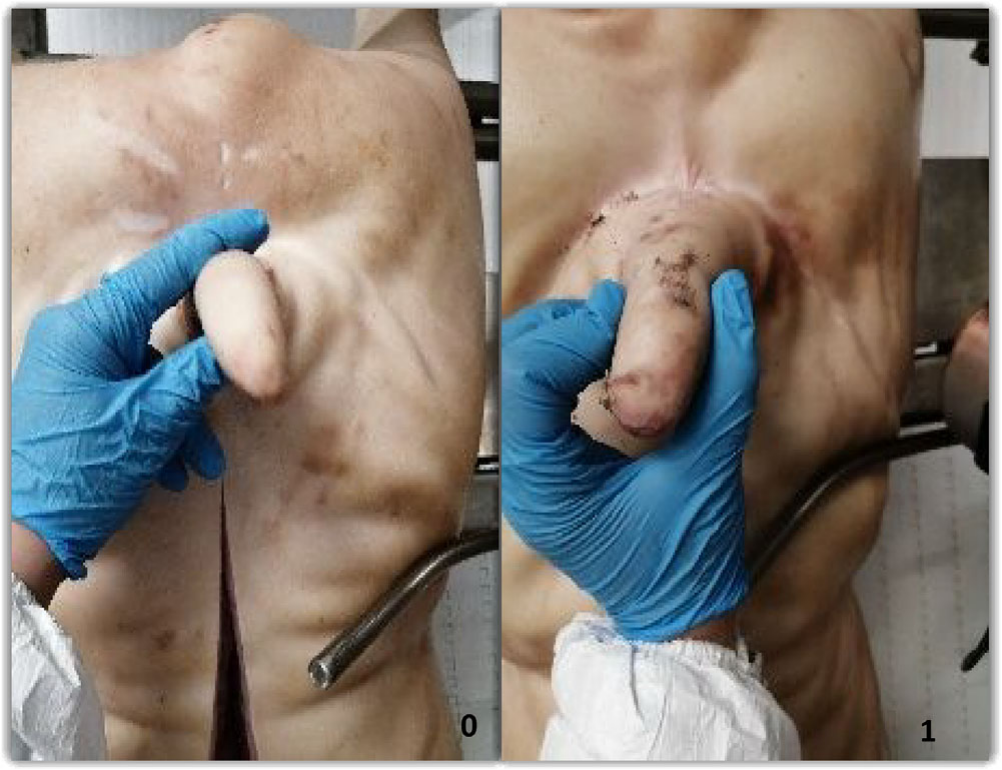
Bruises detection scoring system. 0: absence of bruises; 1: presence of bruises.

The presence or absence of bruising was recorded using a two‐level scoring method: 0—absence of bruises; 1—presence of bruises. The presence of bruises was defined as a darker coloured area on the skin of the tail (Figure 2).

### Statistical analysis

All statistical analyses were performed using SAS 9.3 (SAS Institute Inc., Cary, NC, USA). Descriptive statistics for all VIS and HAND scores were generated using PROC UNIVARIATE. The PROC FREQ function was used to assess the frequency of agreement between scores for each method. Intra‐observer reliability was determined using a Spearman correlation and Cohen's kappa coefficients (*κ*) using PROC CORR and PROC FREQ, respectively. The association between the degree of tail skin damage and the presence of bruises was also evaluated using the Spearman correlation and the chi‐square test. The latter was only used on the data collected while using HAND because we considered this to be the gold standard. Two cut‐off values were applied: (1) the presence of any level of tail skin damage compared with no damage—score 0 versus all other scores and (2) the presence of moderate and severe damage, relative to no or minor damage—scores 0, 1 and 2 compared with score 3 or greater.

### Technical performance and accuracy of the systems (sensitivity and specificity)

To evaluate the technical performance of VIS relative to HAND, sensitivity (SE), specificity (SP), accuracy (AC), positive predictive value, negative predictive value, positive likelihood ratio and negative likelihood ratio tests were calculated using HAND as the gold standard with the same two cut‐off values as previously described. The latter represents the type of lesions typically identified by the veterinary inspectors at the postmortem examination.^21^, ^22^ Similarly, the same tests were also used to estimate the detection of bruises on pigs’ tails. Confidence intervals (CIs) for SE and SP were calculated using the exact (Pearson–Klopper) method, while CIs for predictive values were based on the logit method.^23^, ^24^

### Impact of batch level prevalence on performance of visual inspection

Given that misclassification may occur at different rates with affected (false negatives) and unaffected (false positives) cases, the degree to which apparent prevalence (AP) under‐ or overestimates the true prevalence (TP) will vary according to the TP of tail lesions in the batch of pigs assessed. To demonstrate, three illustrative TP scenarios (0.25, 0.5, 0.75) were simulated. The resulting AP was calculated based on the SE and SP calculated using both cut‐off values, using the following equation: AP = TP × SE + (1 – TP) × (1 – SP).

## RESULTS

Batches ranged in size from 79 to 568 tail‐docked pigs (109.89 ± 71.39). The percentage of animals with severe tail loss with healing was 1.33%. The majority of tails (99.8%) were docked, which in Ireland typically involves removal of one‐half to two‐thirds of the tail.

**FIGURE 3:**
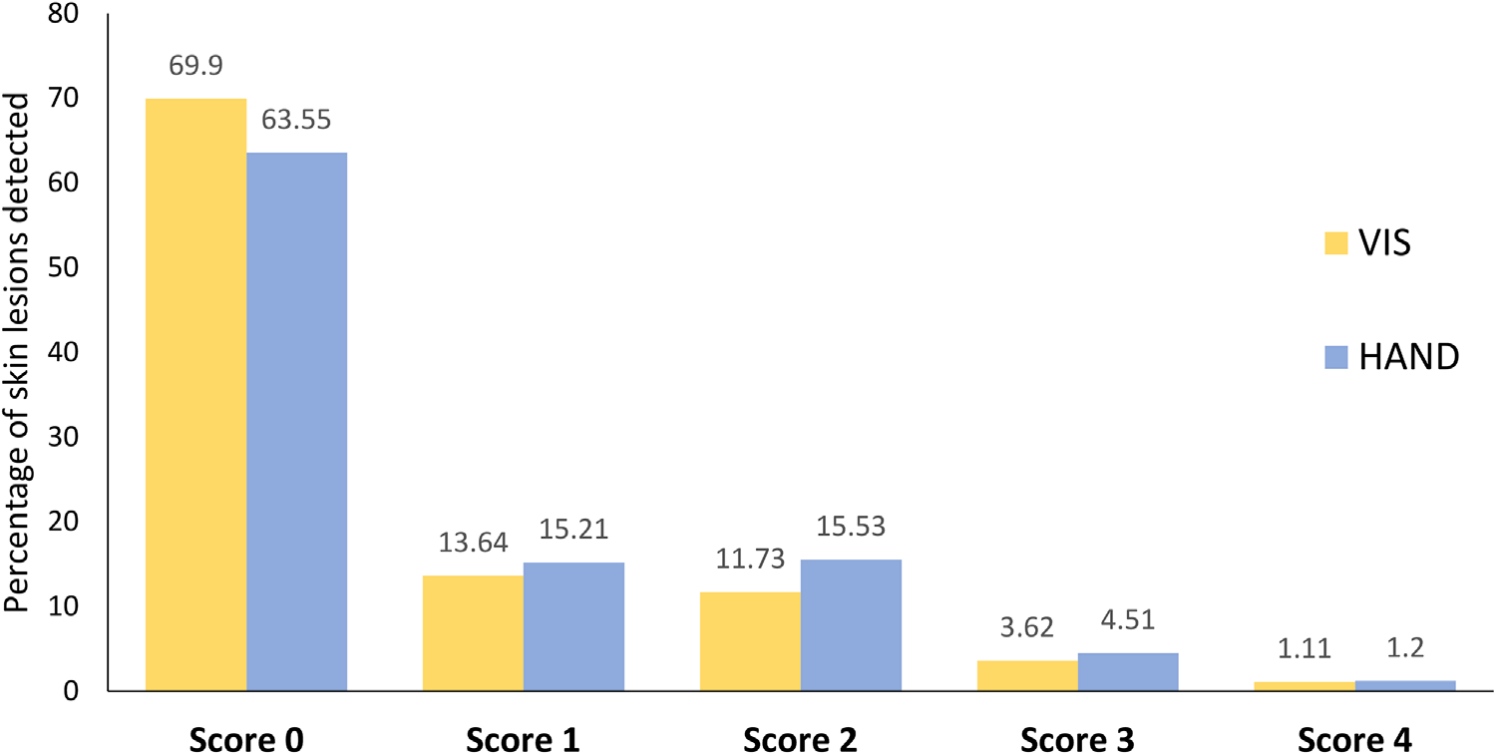
Percentage of tail lesions detected using both visual (VIS) and handling (HAND) systems over a total of 5498 pig carcases examined postmortem.

With regard to the tail skin damage scoring system, we observed variation in the proportion of tail lesions falling within each of the scores depending upon the scoring method. Undamaged skin, score 0, was the most prevalent outcome recorded using both methods. We recorded a higher proportion of mild lesions (scores 1 and 2) using HAND than VIS (Figure 3). The scores assigned using both methods were highly correlated (Table 1). The percentage of agreement between methods was 90.5% and the weighted kappa coefficient was 0.816.

**TABLE 1:** Spearman correlation, level of agreement between systems and Cohen kappa coefficient (score 0–4, no tail lesion to severe, *n* = 5498) for visual (VIS) versus handling (HAND) methods of scoring for both skin damage and presence of bruises.

	Spearman correlation (*ρ*)	Agreement between methods	Cohen's kappa (*κ*)
Tail damage	0.84	90.5%	0.82
Bruises	0.79	89.9%	0.78

**FIGURE 4:**
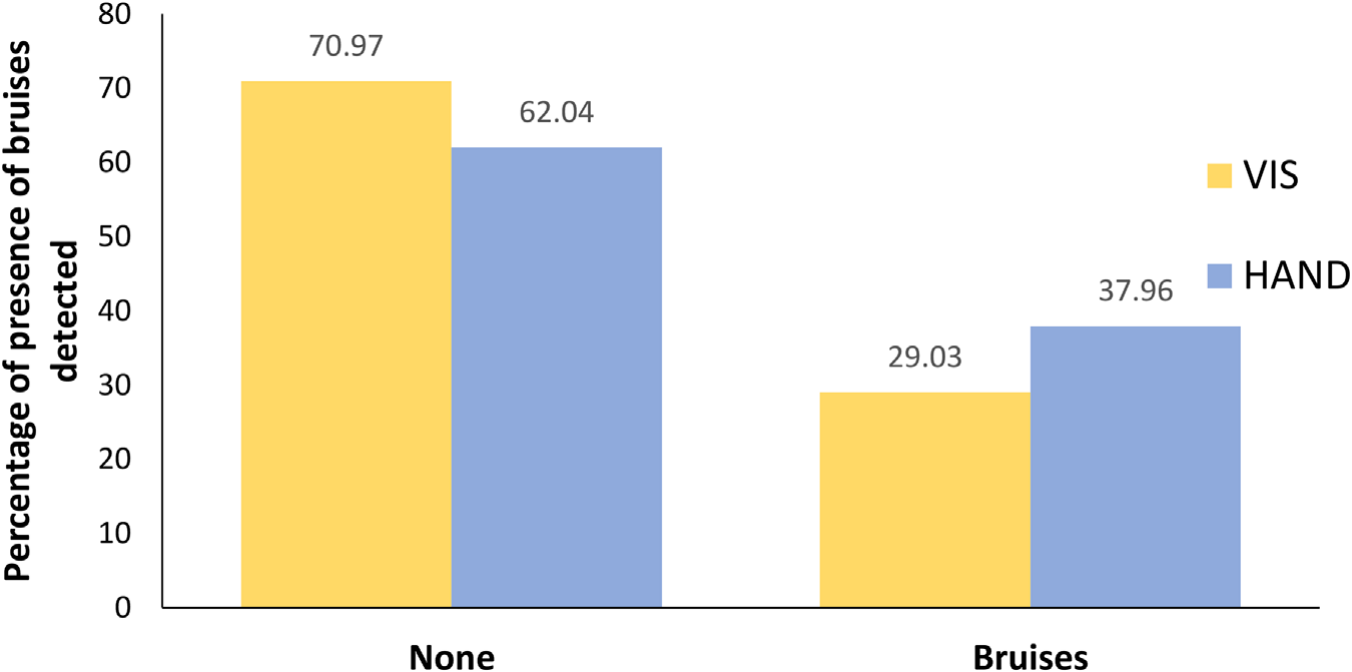
Percentage of presence of bruises detected using both visual (VIS) and handling (HAND) systems in 5498 pig carcases examined postmortem.

Figure 4 shows the variation between the scoring systems with regard to presence or absence of bruises observed. The HAND method detected a higher percentage of bruises than the VIS method, 37.96% and 29.03%, respectively. The agreement between methods for bruise detection was 89.9%. Spearman's correlation between scoring methods was 0.792 and the weighted kappa coefficient was 0.776 (Table 1).

### Technical performance and accuracy of the scoring systems (sensitivity and specificity test)

Using the HAND examination as the gold standard, Table 2 reports the results of the SE and SP tests and the AC of VIS assessment. Given the definition as the proportion of tails with skin damage, the relative SE obtained for the first cut‐off value of score 0 versus all other scores was 80.64% (95% CI: 78.8%–82.3%). The SE for the second cut‐off point of scores 0, 1 and 2 compared with scores 3 and 4 was 82.48% (95% CI: 77.9%–86.3%). The SP, defined as the proportion of tails without skin damage correctly categorised, was 99.98% in both case scenarios (95% CI: 98.5%–99.2% and 99.9%–1%, respectively). The same analysis was performed for the presence of bruises, where the SE (proportion of tails with bruises correctly categorised) was 74.98% (95% CI: 73.1%–76.8%) and SP (proportion of tails without bruises correctly categorised) was 99.09% (95% CI: 98.7%–99.3%).

**TABLE 2:** Calculated measures of accuracy for skin damage and presence of bruises for 5498 pig carcases according to the two cut‐off points used.

	Sensitivity (%)	Specificity (%)	Positive likelihood ratio	Negative likelihood ratio	Positive predictive value (%)	Negative predictive value (%)	Accuracy (%)
Tail damage^a^	80.64	98.88	72.24	0.20	97.64	89.90	92.23
Tail damage^b^	82.48	99.98	4275.97	0.18	99.62	98.95	98.98
Bruises	74.98	99.09	82.51	0.25	98.05	86.62	89.94

^a^ Cut‐off value: any level of damage relative to no damage—score 0 versus all other scores.

^b^ Cut‐off value: no and mild damage versus moderate and severe damage—scores 0, 1 and 2 versus scores 3 and 4.

### Impact of batch level prevalence on performance of visual inspection

Table 3 shows the AP of lesions, based on the three illustrative cases of TP, and the SE and SP results calculated using the two cut‐off thresholds mentioned above.

**TABLE 3:** Expected apparent prevalence of skin damage determined by visual assessment using three illustrative true prevalence scenarios.

	Expected apparent prevalence of damages	
Simulated true prevalence	Lesion present versus absent	No/mild lesion versus moderate/severe lesion
0.25	0.203	0.206
0.50	0.405	0.412
0.75	0.606	0.618

*Note*: The expected apparent prevalence was calculated using the sensitivity and specificity values for of visual assessment, using two cut‐off values: any level of damage relative to no damage—score 0 versus all other scores; no and mild damage versus moderate and severe damage—scores 0, 1 and 2 versus scores 3 and 4.

### Correlation between tail skin damage and bruises

**FIGURE 5:**
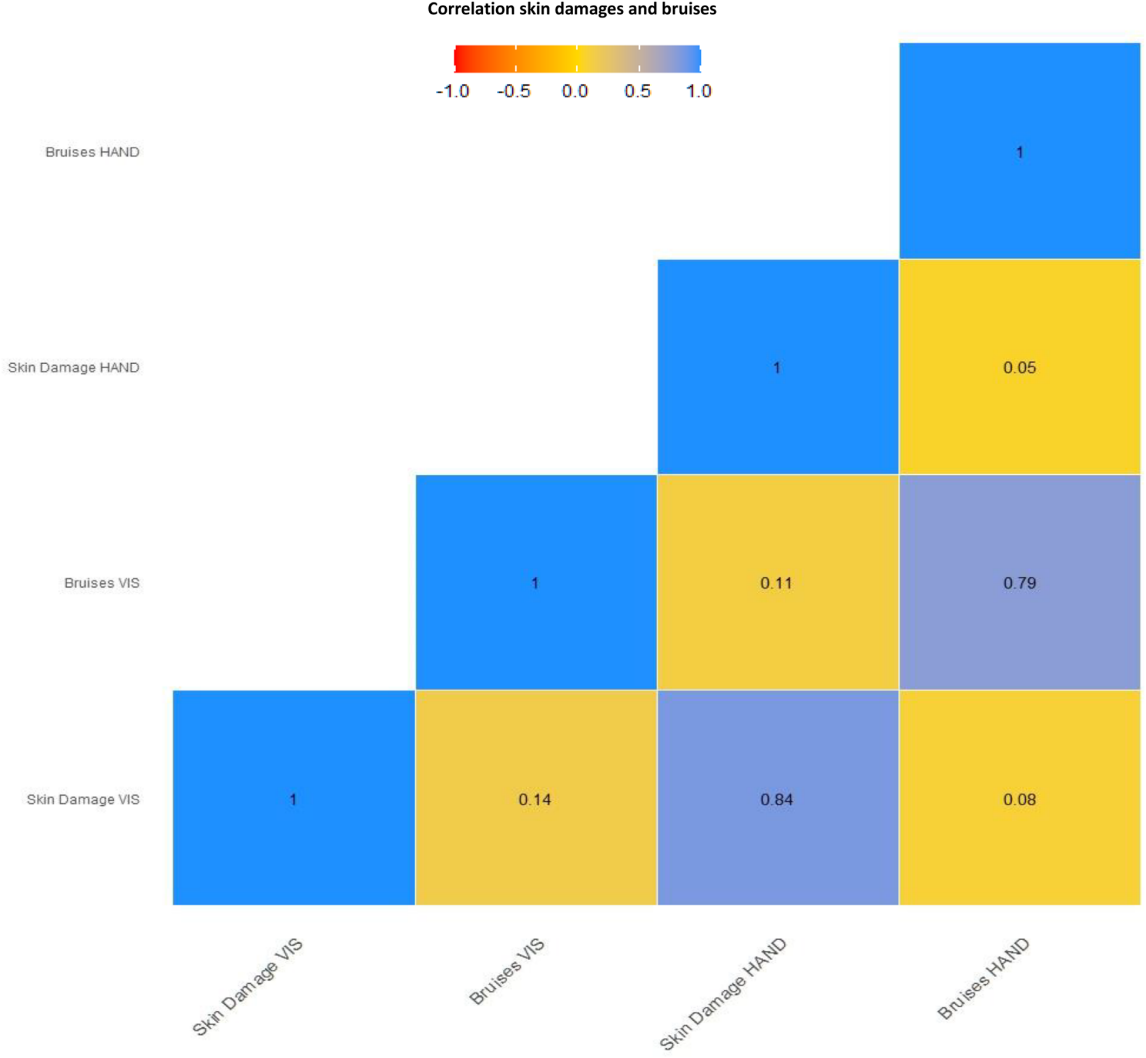
Spearman correlation between skin damages (score 0–4, no tail damage to severe, *n* = 5498) and bruises (score 0–1, presence or absence) detected while using visual (VIS) and handling (HAND) assessments (pattern in yellow).

Tail skin damage and bruises were positively but weakly correlated with each other, whether using the VIS (*ρ* = 0.144; *p* < 0.0001) or HAND (*ρ* = 0.05; *p* < 0.001) methods (Figure 5). The chi‐square test demonstrated that there was strong agreement between the presence of tail lesions and the presence of bruises (*χ* ^2^ = 20.97; *p* < 0.001). Similarly, there was an association between the presence of bruises and moderate and severe lesions, although this was not as strong (*χ* ^2^ = 2.51; *p* < 0.5).

## DISCUSSION

We hypothesised that VIS examination of pigs’ tails could be a valid substitute for identifying skin damage and bruising compared to traditional HAND examination. We also hypothesised that scoring bruises independently from tail skin damage could enhance the information gleaned from VIS where the inability to manipulate the tails could lessen the chance of detecting mild skin damage. While our results in general supported our hypotheses, it is important to acknowledge the limitations of the study. All tails were scored using VIS and HAND in quick succession and always in that order by the same observer, which potentially impacted the independence of the two variables. Visual scoring was carried out first to minimise bias given the potential influence of knowing the HAND score.

We tested our hypotheses and came to this conclusion by using a range of tests. The results show that VIS alone can correctly identify tail damage caused by tail biting, mainly moderate and severe injuries, which are arguably the more important lesions to detect. Furthermore, we found a high level of agreement and AC between the tests. In addition, we identified a correlation between the presence of injuries and bruises. Importantly, this confirms that the bruises probably had the same underlying causes as the lesions/injuries, such as tail manipulation by another pig.

The condition of pigs’ tails at slaughter is an ‘iceberg’ indicator of pig welfare on farms.^12^, ^25^ Thus, investigation of methods to improve and ease collection of this type of data is important. The use of VIS examination has potential to reduce workload for the operator and facilitate scoring when the line speed is quick or for tails that are out of reach. A few studies assessed the use of a camera to automatically detect lesions on pig carcasses at slaughter and found that it was comparable to the traditional inspection method.^19^, ^26^ However, to the best of our knowledge, this is the first study to focus on the comparison between methods to score skin damage on pigs’ tails, comparing the traditional methods of manipulating part of the carcase (HAND) and a visual inspection by a person (VIS). It is possible that the relationship between HAND and VIS carried out ‘in‐person’ may be closer than the relationship between traditional HAND and a camera, which adds another layer of uncertainty, such as differences between the camera's ability to detect colours and the human eye. Additionally, we are unaware of previous studies regarding the detection of bruises independent of other tail lesions at slaughter, let alone the most effective way to assess them at postmortem examination. Our results demonstrated that scoring both tail skin damage and bruises is possible using VIS scoring, based on the frequency of lesions and bruises detected and their severity.

In this study, we detected skin damage on the tails of approximately one‐third of pigs, whether using VIS or HAND. This did not include the percentage of severe tail lesions with healing, which was detected in just over 1% of carcases, using both methods. A severe tail lesion with healing consisted of complete tail loss with evidence of scar tissue and it was detectable visually, as there was no tail present to handle. This parameter indicates a history of severe tail biting on the farm, even though the biting may not have happened near the time of slaughter. If this were the case the skin would not yet have fully healed and would be represented by a score of 4 in the scoring system. Thus, this type of scarring might be an indicator of biting that occurred earlier in the production cycle and could be a very important measure to include if recording tail condition at slaughter.^27^ Further research should aim to investigate whether there is a link between severe tail biting at weaner stage and scars or signs of healing evident on the carcase.

Our results revealed that the detected prevalence of all five scores relating to skin damage (scores 0–4) did not vary remarkably between the two methods of assessment. In general, the VIS system resulted in the observer detecting a slightly higher percentage of intact skin of pigs’ tails but fewer mild lesions compared to HAND. The location of the lesions can provide a possible explanation. With VIS, the observer can only investigate one side of the tail. For VIS examination of a long tail, the observer can visualise the ventral section from the base to the tip; meanwhile, for docked tails, the visualisation of the tail can be either ventral or dorsal, depending on the position of the observer. The tail manipulation that occurs using HAND, such as rotating and pulling, facilitates a clearer view of the entire tail because it allows the examination of parts of the skin otherwise not visible. It is also possible that the VIS system could lead to the misclassification of tail lesions such as skin breakage derived from the scalding and dehairing process. Such misinterpretation is less likely if the tail is manipulated.

Machinery‐related damage to the carcass can manifest as shredding and peeling of the skin, creating lesions that lack colour, which indicates that they occurred after exsanguination. In the experience of the authors, tail skin damage due to biting is often associated with a reddish colour (even in mild cases) and, depending upon the severity of the lesion, presents visible bite marks or (in the case of healed tail lesions) significant scar tissue.

When considering moderate (score 3) and severe (score 4) tail lesions, the prevalence results were similar using both assessment methods. These lesions are so severe and extensive that they are immediately obvious to the eye, so manipulation of the tail is not necessary. EFSA reported that only severe lesions are routinely recorded in the abattoir.^27^, ^28^ This may explain why the prevalence of tail lesion results recorded by meat inspectors is generally lower compared to results obtained while scoring pigs’ tails on the farm,^29^ or by studies at the abattoir,^30^ because minor lesions are included. Our results, comparing HAND and VIS inspections, were similar to a previous study where the assessment of moderate and severe lesions showed a better validity and repeatability than assessing mild lesions.^31^ Moreover, the outcome obtained by the statistical analysis confirmed that a VIS examination to detect tail lesions was a reliable measure for a postmortem examination in the abattoir. However, when analysing the AP based on three possible case scenarios, our results showed that the higher the actual prevalence of lesions, the greater the likelihood that the levels of tail damage were underestimated when VIS is used.^32^

The finding that the percentage of tail bruises detected was higher using HAND than VIS could also be explained by the carcass processing method in the abattoir. It was hypothesised that the scalding and de‐hairing process of the carcass could impact tail lesion scores in a number of ways.^30^ First, bruises might become more visible after the process due to the tails being cleaned. However, it is also possible that small bruises derived from tail biting could be misjudged for a dark spot derived from the scalding process. Moreover, the singeing process could result in red–brownish marks on the tail.^30^ The author's observation was that when closely observed, bruises on pigs’ tails were characterised by a colour variation: going from black/dark blue at the centre to slightly blue–brown towards the edge of the lesion. This close examination required manipulation of the tail of the carcass and was not always detectable by VIS. Additionally, smaller bruises were not detectable when using a VIS scoring method. These observations are in line with a previous study that suggested that singeing was responsible for disguising pre‐existing inflammatory changes in the subcutaneous tissue of the tail.^13^ Nevertheless, while the ability to palpate or manipulate pigs’ tails is still an important factor in detecting the presence of bruises, the level of agreement between HAND and VIS in our study was high, indicating that the VIS could be used to replace the HAND system on the slaughter line.

The present study found a slight positive correlation between skin damage and bruising on pigs’ tails. Indeed, there was a strong association between the presence skin damage and the presence of bruises. This suggests that the underlying cause for both forms of damage could be related. Interestingly, the association was stronger when mild skin damage (score 1) was used as the threshold for comparison, rather than only using moderate to severe skin damage. It is possible that this type of injury could be associated with ‘tail in mouth’ behaviour, which involves a pig manipulating another pigs’ tail in its mouth gently, rather than biting and often does not break the skin. It is thought that this type of behaviour could act as a precursor to severe tail biting outbreaks; as such, it could be an indicator of low level, ongoing biting, that could pose a risk for more serious injuries. Indeed, other studies also suggest that bruising is a consequence of tail bites.^11^, ^33^ Another study reported an association between bruises and bite marks and healed tails and a higher level of skin infection; however, bruises were scored within the same scoring system as minor breaks to the skin and, as such, were not considered independently.^13^

There may be other factors that trigger the possibility of an inflammatory cause leading to the presence of bruises, such as antemortem handling or animal transport.^13^ Our results showed less of an association between moderate/severe tail skin damage and the presence of bruises than between any level of skin damage and bruises. We recommend that skin damage and bruising on the tail should ideally be scored separately to provide an additional layer of information as to what could be the primary cause. In general, visual inspection resulted in fewer lesions identified, mild to severe, and a lower percentage of bruises identified.

This study showed that inspection of tails solely by a VIS method when using a well‐defined protocol for evaluating tail skin damage and bruising could be a feasible alternative to handling parts of the carcass. This could lower the risk of contamination of carcasses and spreading bacteria. This is also of interest to researchers who want to evaluate possible relationships between injuries and bruise formation in the surrounding areas. Further research should investigate the observer's best positioning on the slaughter line and the relationship between the length of the tail and presence of tail skin damage. This study also demonstrated the potential for automated scoring of tails, as VIS scoring was a good alternative to HAND. The position of tools, such as a camera or a computerised system, may remove the need for a human observer and should be investigated further.

## AUTHOR CONTRIBUTIONS

Roberta Maria D'Alessio and Keelin O'Driscoll contributed to the conception and design of the study. Laura Ann Boyle contributed to training the first author on scoring pigs’ tails in the slaughterhouse. Roberta Maria D'Alessio, Keelin O'Driscoll and Conor G. McAloon performed the statistical analysis. Roberta Maria D'Alessio planned and organised the article and drafted the manuscript. Alison Hanlon, Keelin O'Driscoll, Laura Ann Boyle and Conor G. McAloon revised the manuscript. All authors contributed to the article and approved the submitted version.

## CONFLICTS OF INTEREST STATEMENT

The authors declare they have no conflicts of interest.

## ETHICS STATEMENT

No ethical proposal was submitted for the study.

## Data Availability

The data that support the findings of this study are available from the corresponding author upon reasonable request.
